# The Effects of Needling Fenglong (ST40) and Neiguan (PC6) on IL-17 of ApoE-Gene-Knockout Mice's Liver

**DOI:** 10.1155/2014/691863

**Published:** 2014-03-20

**Authors:** Fu Yun Lee, Ze Jun Huo, Li Zhang, Jia Guo, Huan Chen, Tong Liu, Bo Peng, Pei Xin Hong, Yuan Yuan Peng, Yi Fan Fan, Yu Pei Chen

**Affiliations:** ^1^School of Acupuncture and Moxibustion, Beijing University of Chinese Medicine, No. 11 North 3rd Ring Road, Chaoyang District, Beijing 100029, China; ^2^Acupuncture and Moxibustion Department, The Third Hospital of Peking University, No. 49 North Garden Road, Haidian District, Beijing 100191, China

## Abstract

The aim of the present paper was to observe the effects of needling ST40 and PC6 on IL-17 of ApoE^−/−^ mice with fatty liver. Forty male ApoE^−/−^ mice were randomized into Needling-Acupoint Group, Simvastatin Intragastric Administration Group, Needling Nonacupoint Group, and Model Group. Each was fed with high fat diet for 8 weeks since 16 weeks of age; after 8 weeks of intervention, mice were sacrificed and tested for various examinations. Result showed that the body weight, TC, and serum IL-17 in Needling-Acupoint Group decreased. Compared with Model Group, the immunohistochemical expressions of IL-17 in liver tissue were significantly decreased among the other three groups. In conclusion, acupuncture was able to lower the expression of IL-17 level both in serum and liver tissue in ApoE^−/−^ mice, which is helpful to reduce the inflammation and defers the progress from fatty liver to cirrhosis.

## 1. Introduction 

As people's living standards improved, the intake of protein, fat, and alcohol has largely increased, which might lead to metabolic disorders and hyperlipidemia and trigger fatty liver disease. It is estimated that there might be over 160 million adults suffering from dyslipidemia in China. However, long-term use of lipid lowering drugs may result in side effects such as hepatic and renal dysfunction, which endanger human health [1]. The research [2–4] indicated that acupuncture was able to reduce blood lipids which, with fewer side effects, were safer.

The inflammatory cytokines inside and outside the liver play a critical role in chronic liver diseases, including fatty liver. The significant increase of IL-17 (interleukin, IL-17) can be detected in hyperlipidemic fatty liver and also cirrhosis patients [5]. IL-17 is able to promote the release of kinds of cytokines which are involved in inflammatory diseases such as IL-6, which forms a positive feedback with IL-17 [6, 7]. If the hyperlipidemic fatty liver is not well controlled, the inflammation exacerbates, then it might develop into hepatitis, cirrhosis, cardiovascular, and cerebrovascular diseases or other liver and kidney diseases [8, 9].

In this study, from inhibiting the activity of inflammatory cytokines, we analyzed the effects of acupuncture on IL-17 expression in fat excess liver and provided some basic evidences that the inflammatory damage of hyperlipidemic fatty liver could be restricted through acupuncture.

## 2. Materials and Methods

### 2.1. Materials

*Experimental Animal.* Adult male ApoE-gene-knockout mice (16 weeks of age, 23.6 g–30.5 g) were purchased from Vital River Laboratory Animal Technology Co. Ltd., batch number: SCXK (Beijing) 2011-0012.

*Needling Instrument and Reagent.* HuanQiu acupuncture needle, 0.20 × 20 mm, batch number: LOT/BATCH, (Suzhou Acupuncture Goods Co., Ltd.). Simvastatin, (Hangzhou MSD Pharmaceutical Co., Ltd.). Anti-IL-17 antibody (Abcam, UK).

### 2.2. Methods

#### 2.2.1. Grouping Experimental Animals

After normal diet feeding for one week, the ApoE^−/−^ mice were randomly and equally divided into four groups: Needling-Acupoint Group; Simvastatin Intragastric Administration Group; Needling Nonacupoint Group, and Model Group. They were kept in SPF class experimental animal room, with temperature 23 ± 2°C, relative humidity 60–65%, a 12-hour light-dark cycle (7:00 am–7:00 pm), and free access to water and food.

#### 2.2.2. Model Preparation

After grouping, mice were fed with a high-fat diet, containing 21% fat, and 0.15% cholesterol supplied by Department of Laboratory Animal Science at Peking University, China.

#### 2.2.3. Processing Methods

Mice in Needling-Acupoint Group were received acupuncture at both sides of ST40 and PC6 with 20 mm needles in diameter. ST40 was performed by straightly inserting a stainless steel needle to a depth of 3 mm and PC6 was obliquely toward the elbow to a depth of 2 mm. The needles were rotated slowly at the speed of 60 rounds per minute to moderate reinforcing and reducing. The entire procedure was completed in 2 minutes without retaining needle, three times a week for 8 weeks. The Simvastatin Intragastric Administration Group received Simvastatin intragastric administration (25 mg/kg/d) for 8 weeks. Needling Nonacupoint Group received nonacupoint needlings (two points in 0.5 cm and 1 cm to the end of tail), each inserted obliquely 1 mm in depth. Mice in Model Group were tied up without acupuncture and bred normally as the other groups.

#### 2.2.4. The Measurements

1. Body weight: measuring body weight before and after the experiment.
2. Serum indicators: TC was detected at the beginning and the end of the experiment and IL-17 was also tested by ELISA.
3. Histopathological examination: some fresh liver tissue was made into frozen section and stained with Oil-Red-O staining as well as Haematoxylin and eosin staining to observe the degree of hepatic steatosis.
4. Immunohistochemical method for the expression of IL-17 in liver tissue. Three portal areas were selected randomly in each staining section and their positive expressions in cytoplasm were assessed by IOD [10].

### 2.3. Statistical Processing

SPSS17.0 software was employed. Comparisons between groups were analyzed by One-Way ANOVA and LSD test. The Data of each group were expressed as mean ± SD, *P* < 0.05 for statistical significance, and *P* < 0.01 for a significant difference.

## 3. Result

### 3.1. Body Weight Decreased Significantly in Needling-Acupoint Group

In first week of the experiment, the body weight among 4 groups was not significantly different (*P* > 0.05). After 8 weeks of intervention, compared with Model Group, the body weights of Needling-Acupoint Group and Simvastatin Intragastric Administration Group decreased (*P* < 0.05); The body weight in Needling Nonacupoint Group rose slightly (*P* > 0.05).

### 3.2. Serum TC and IL-17 Decreased in Needling-Acupoint Group

There was no distinguished difference of TC among 4 groups before the experiment (*P* > 0.05). After 8 weeks intervention, compared with Model Group, TC of the Needling-acupoint Group and Simvastatin Intragastric Administration Group were lower, but only the Needling-acupoint Group was statistically significant (*P* < 0.05), and the Needling Nonacupoint Group decreased little. (*P* > 0.05, Table 2). In comparison with model group, serum IL-17 of Needling-acupoint Group and Simvastatin Intragastric Administration Group were significantly lower (*P* < 0.01), while Needling Nonacupoint Group went down lightly, with no statistical significance (*P* > 0.05, Table 2).

### 3.3. The Pathological Changes of Hepatic Tissues

Frozen sections of liver tissue were prepared for Haematoxylin and eosin staining and Oil-Red-O staining. Under the microscope, frozen sections showed that the liver tissue in Model Group grew varied hepatic steatosis, such as enlarged hepatic cells, structural disorder, and many lipid droplet vacuoles within the cytoplasm. By Oil-Red-O staining, numerous deep dyeing and large lipid droplets within cytoplasm can be seen.

In Needling Nonacupoint Group, the steatosis appeared and the enlarged hepatic cells are similar to that of Model Group. There were many deep dyeing lipid droplets in portal areas.

In Needling-acupoint Group, after acupuncture treatment, steatosis of the liver tissue has significantly alleviated and its structure tended to be normal, though, only a few scattered small dyeing lipid droplets in liver cells can be seen.

**Figure 1:**
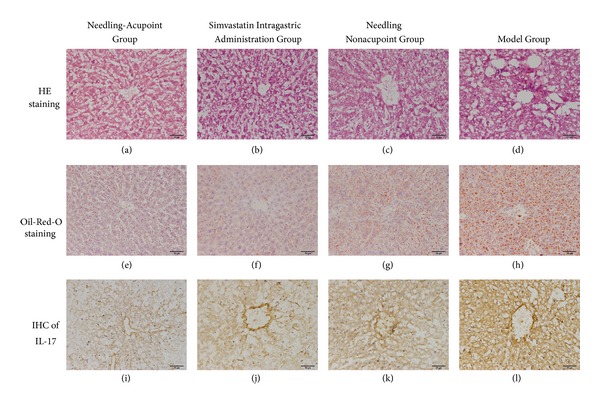
Histological observation of liver tissue in each group.

Mice in Simvastatin Intragastric Administration Group also had more regular liver cell structure than Model Group. There were Oil-Red-O stained lipid droplets varied in number and size around the portal areas, which were smaller, lighter, and less compared with those of Model Group (Figure 1).

### 3.4. Immunohistochemistry of IL-17 Expression in Liver

Immunohistochemical results showed that the Model Group had strong IL-17 positive cells and brown pigmentation particles, deep in color and large in size. The pigmentation particles in Needling-acupoint Group and Simvastatin Intragastric Administration Group were lighter and smaller compared with Model Group. Unlike Model Group, the particles in Needling Nonacupoint Group were light colored, yet darker than those in Needling-acupoint Group and Simvastatin Intragastric Administration Group (Figure 1). The positive regions were measured to assess IOD with Image Pro Plus 6 software (Table 1), and the IOD of the other three groups lowered significantly than Model Group (*P* < 0.01), with no significant difference among the three.

## 4. Discussion 

Four of the findings of acupuncture on ApoE^−/−^ mice' ST40 and PC6 are worth summarizing. (1) The body weight decreased, (2) TC and serum IL-17 lowered, (3) pathological changes in hepatic tissues improved, and (4) immunohistochemical expression of IL-17 in liver significantly reduced.

The term “Fatty Liver” does not exist in Traditional Chinese Medicine, but its syndrome relates to accumulation, distention of abdomen, jaundice, hypochondriac pain, turbid phlegm, and so on, involving phlegm, dampness, blood stasis, and mass. Although the disease locates in the liver, the spleen and kidney are also related, and its first pathogenesis is the deficiency of spleen and kidney. The disease is caused by overeating greasy and sweet food and drinking excessively or by invasion of damp-heat epidemically exogenous pathogen, mental disturbance, and long illness, which could lead to the liver failing to maintain the normal flow of Qi, the spleen failing to transport and convert, and phlegm stasis. Furthermore, the kidney deficiency develops, so do the phlegm and blood stasis, afterwards the disease is formed. The treatment mainly focuses on smoothing the liver to strengthen the spleen, reducing phlegm to eliminate dampness, and on eliminating blood stasis to activate blood circulation, concurrently reinforcing the liver and kidney [2, 11–13].

Treating hyperlipidemic fatty liver with acupuncture, its mechanism operates in inhibiting activity of inflammatory factors besides improving insulin resistance, antioxidative stress [14, 15]. In this experiment, we chose Fenglong (ST40) and Neiguan (PC6) to treat fatty liver. As a key acupoint to deal with phlegm, ST40 is able to communicate Stomach Meridian Foot-Yangming (ST) and Spleen Meridian of Foot Taiyin (SP). The point functions in regulating spleen and stomach, clearing down phlegm, activating channels, and reducing tangible or intangible phlegm. As for PC6, it belongs to Pericardium Meridian of Hand-Jueyi, when compatible with ST40, it would tranquilize the mind, relieve the pain, regulate Qi flow and stomach, nourish the blood, promote blood circulation, and clear down phlegm [16–18].

Liver, as an important organ for lipid metabolism, centers on fat intake, oxidation of fatty acid, and the synthesis and secretion of cholesterol, phospholipid, and lipoprotein. When the lipid metabolism disorders, a large amount of fat enters into hepatocytes, which increases fat synthesis. If this grows beyond the hepatocytes' capability in Oxidation and Synthesis of lipoprotein, the lipids have to accumulate in the liver cells, leading to denaturing and swelling of the liver cell, consequently the inflammation, necrosis, and fibrosis of fatty liver. Hyperlipidemia shoulders large part in the formation of fatty liver, which means that it presents the positive correlation to the morbidity of fatty liver. Inflammatory factors inside or outside the liver play crucial role in the incidence of fatty liver and they are part of the early manifestations of the metabolism disorder.

IL-17 could be interpreted as the inflammatory factors mainly produced by CD4^+^ T lymphocyte subsets (Th17). This kind of proinflammatory factors has strong induction on neutrophils and simultaneously promotes the expressions of various cytokines, such as the expression and release of IL-6, IL-18, and TNF-*α*, various inflammatory diseases in human body are also related to them [19]. Th17 and IL-17 can also accelerate the progress from simple fatty liver to nonalcoholic steatohepatitis (NASH) [20, 21].

In this study, ApoE^−/−^ mice were used as the model of hyperlipidemic fatty liver, and ST40 and PC6 were acupunctured. It showed that acupunctural intervention on ApoE^−/−^ mice would decrease IL-17 expression in serum and liver tissue. Likewise, serum total cholesterol was decreased. This result echoes Li Li Zhu et al.'s [3] and Li Zhou et al.'s [4] finding that acupuncture is capable of reducing TC in mice and rats. Although there have been many studies on observation of the impact of IL-17 on various diseases, few have been made on IL-17 control of fatty liver disease. On this basis, it can be believed that other inflammatory factors might also be reduced, for instance, inhibiting the positive-feedback loop produced by IL-6 [22]. With needling the nonacupoints, expression of IL-17 in liver tissue also could be reduced. Therefore, it could be concluded that acupuncture is helpful to reduce the hepatic inflammation and to slow down the speed of fatty liver developing into hepatitis or cirrhosis.

## 5. Conclusion

Needling on ST40 and PC6 of ApoE^−/−^ mice is capable of lowering TC and might also be able to control the expression of IL-17. In spite of all the limitations of our conclusions, in order to obtain more reliable and objective data, further research is required in a number of directions. For instance, on the topic about the effect and mechanism of regulating lipid metabolism by acupuncture at single acupoint, how IL-17 varies in tissue or serum at different time or the IL-17 involved signaling pathways. Hopefully, future study can not only provide a better understanding of acupoint specificity, but also reflect the development of disease, through immune regulation to guide the treatment.

## Figures and Tables

**Table 1:** Body weight and IOD (*n* = 8).

Group	Weight (g)		IOD
	Week 1	Week 8	
Needling-Acupoint Group	27.78 ± 0.85	26.37 ± 1.50^a^	6.75 ± 2.34^b^
Simvastatin Intragastric Administration Group	27.90 ± 1.47	26.87 ± 1.35^a^	6.16 ± 1.61^b^
Needling Nonacupoint Group	27.62 ± 1.28	29.12 ± 1.95	6.54 ± 0.91^b^
Model Group	26.68 ± 1.19	28.87 ± 2.35	9.54 ± 2.70

IOD: Integrated optical density; Note: compared with model group,  ^a^ *P* < 0.05,  ^b^ *P* < 0.01.

**Table 2:** Serum TC and IL-17 (*n* = 8).

Groups	TC (mmol/L)		Serum IL-17 (umol/L)
	Week 1	Week 8	
Needling-Acupoint Group	10.80 ± 2.64	19.84 ± 4.23^a^	25.49 ± 4.35^b^
Simvastatin Intragastric Administration Group	9.62 ± 1.41	20.89 ± 2.84	24.14 ± 6.81^b^
Needling Nonacupoint Group	11.39 ± 1.17	22.68 ± 4.53	40.56 ± 5.91
Model Group	10.34 ± 2.91	24.15 ± 4.00	43.49 ± 5.46

TC: Total cholesterol; Note: compared with model group,  ^a^ *P* < 0.05,  ^b^ *P* < 0.01.
